# Behavioural support and nicotine replacement therapy for smokeless tobacco cessation: protocol for a pilot randomised-controlled multi-country trial

**DOI:** 10.1186/s40814-022-01146-5

**Published:** 2022-08-22

**Authors:** Faraz Siddiqui, Linda Bauld, Ray Croucher, Cath Jackson, Ian Kellar, Mona Kanaan, Subhash Pokhrel, Rumana Huque, Romaina Iqbal, Javaid Ahmed Khan, Ravi Mehrotra, Kamran Siddiqi, on behalf of the ASTRA Global Health Research Group

**Affiliations:** 1 Department of Health Sciences, University of York, Heslington, UK; 2 Usher Institute and SPECTRUM Consortium, University of Edinburgh, Edinburgh, UK; 3 Valid Research Ltd., Wetherby, UK; 4 School of Psychology, Faculty of Medicine & Health, University of Leeds, Leeds, UK; 5 Health Economics Research Group (HERG), Department of Health Sciences, Brunel University London, Uxbridge, UK; 6 ARK Foundation, Dhaka, Bangladesh; 7 Department of Medicine, Aga Khan University, Karachi, Pakistan; 8 ICMR - India Cancer Research Consortium, New Delhi, India

**Keywords:** Tobacco, Smokeless, Tobacco use cessation, Nicotine, Asia

## Abstract

**Background:**

Smokeless tobacco (ST) is consumed globally by more than 350 million people, with approximately 85% of all users based in South and Southeast Asia. In this region, ST products are cheap and easily accessible. Evidence-based interventions to people quit ST use are lacking. This study aims to test the feasibility of conducting a future definitive trial of ST cessation, using a culturally adapted behavioural intervention, and/or nicotine replacement therapy (NRT) in three South Asian countries.

**Methods:**

We will conduct a factorial design, randomised-controlled pilot trial in Bangladesh, India and Pakistan. Daily ST users will be recruited from primary health care settings in Dhaka, Noida and Karachi. Participants will be individually randomised to receive intervention A (4 or 6 mg NRT chewing gum for 8-weeks), intervention B (BISCA: face-to-face behavioural support for ST cessation), a combination of interventions A and B or usual care (Very Brief Advice - VBA). The participants will provide demographic and ST use related data at baseline, and at 6, 12 and 26 weeks of follow-up. Salivary cotinine samples will be collected at baseline and 26 weeks. The analyses will undertake an assessment of the feasibility of recruitment, randomisation, data collection and participant retention, as well as the feasibility of intervention delivery. We will also identify potential cessation outcomes to inform the main trial, understand the implementation, context and mechanisms of impact through a process evaluation and, thirdly, establish health resource use and impact on the quality of life through health economic data.

**Discussion:**

The widespread and continued use of ST products in South Asia is consistent with a high rate of associated diseases and negative impact on the quality of life. The identification of feasible, effective and cost-effective interventions for ST is necessary to inform national and regional efforts to reduce ST use at the population level. The findings of this pilot trial will inform the development of larger trials for ST cessation among South Asian users, with relevance to wider regions and populations having high rates of ST use.

**Trial registration:**

ISRCTN identifier 65109397

**Supplementary Information:**

The online version contains supplementary material available at 10.1186/s40814-022-01146-5.

## Introduction

Smokeless tobacco (ST) refers to a heterogeneous group of tobacco-containing products that are non-combustible and consumed either orally or by inhalation through the nose [1]. While ST is consumed by more than 350 million individuals worldwide, approximately 85% of users are concentrated in South and South-East Asia [2]. In these low-resource settings where ST is culturally ingrained, it remains cheap, widely available and poorly regulated [3]. These South and South-East Asian ST products often contain stimulants and flavourings that enhance flavour and addictiveness. On the other hand, their preparation methods induce high levels of free nicotine, Tobacco Specific Nitrosamines (TSNAs) and heavy metals [4]. Due to their composition, South and South-East Asian ST products are highly addictive and toxigenic. Their continued use results in oral and oropharyngeal cancers [5], rates of which are the highest in South Asia [6]. ST use in these populations is also linked to ischemic heart disease [7], as well as preterm delivery, stillbirth and low birth weight deliveries [8]. In an effort to reduce the impact of ST use in South Asian populations, the ASTRA Global Health Research group [9] was formed in 2018. This group is conducting a range of studies (focusing on cessation, youth, policy and economics) to understand and address ST use in South Asian settings.

The need for evidence-based guidelines and treatment for tobacco dependence is supported by Article 14 of the World Health Organization’s Framework Convention for Tobacco Control (WHO-FCTC) [10]. Tobacco cessation is also included in WHO’s MPOWER approach, which is a set of six high impact policy measures for reducing tobacco use [11]. In the South Asian context, however, evidence and support for ST cessation remain low. The Global Adult Tobacco Survey (GATS) reports that only 31-57% of ST users in South Asia are advised to quit ST by their health care providers [12]. There is also a lack of evidence for effective and cost-effective interventions to support quit attempts in these individuals. To date, two trials have been conducted in South Asian settings, both in India. The first study was a RCT of varenicline which did not demonstrate any difference in biochemically verified abstinence to ST at 12 weeks when compared to a placebo or reduce the risk for a lapse [13]. The other trial was a cluster RCT of yogic breathing exercises which were delivered through community outreach. The intervention was found to have a small, yet significant effect on cessation rates at 6 months. This trial, however, had limited implications for ST users as it included smokers and dual users of smoking and ST products [14].

The use of behavioural interventions (counselling from a trained adviser, or ‘behavioural support’) has previously demonstrated effectiveness in achieving abstinence among ST users [15]. This evidence base builds upon studies of ST users conducted in Europe and North America. The potential of behavioural interventions to support ST cessation remains largely unexplored in South Asian populations. The potential of behavioural support may further be enhanced with the use of pharmacotherapy such as nicotine replacement therapy (NRT) which can reduce withdrawal symptoms in individuals trying to quit [16]. In a multi-centre, prospective cohort study of South Asian ST users in England, NRT use was reported to be acceptable by ST users receiving cessation support and was associated with higher abstinence at 4 weeks when given alongside a behavioural intervention, as compared to the behavioural intervention alone [17]. It is expected that similar uptake and outcomes may be observed in South Asia. A theory-based intervention ‘Behavioural support Intervention for ST Cessation in South Asian communities’ (BISCA) has previously been developed and tested in a small number of ST users in the UK and Pakistan [18] but has not been evaluated in a RCT or in wider South Asian settings. Establishing an evidence base for BISCA and NRT (separately and in combination) in this pilot randomised study will provide a strong foundation for a future definitive randomised trial of interventions for South Asian ST users and support wider efforts in this area.

### Aims and objectives

We aim to conduct a pilot randomised controlled trial of BISCA and NRT (individually and in combination) to inform the design and implementation of a future definitive trial for ST cessation in South Asian resident populations.

Our primary objectives are to analyse and report data on recruitment, randomisation and retention, data collection and intervention delivery. Our secondary outcomes are identification of potential cessation outcomes for the main trial, an assessment of implementation, context and mechanisms of action through a process evaluation, and establishing health resource use and impacts on the quality of life using health economics data.

## Methods

The protocol for the pilot RCT (v1.2) is registered with the ISRCTN registry (65109397). The protocol manuscript confirms to items recommended in the SPIRIT checklist and CONSORT statement extension for pilot trials [19].

### Trial design

We will conduct a pragmatic, individually randomised multi-country pilot RCT of BISCA and NRT. A preliminary economic assessment and process evaluation will be incorporated. The feasibility trial will use a 2×2 factorial design (Table 1) incorporating the following interventions:

1. Intervention A (4 or 6 mg NRT chewing gum for 8 weeks)
2. Intervention B (BISCA: face-to-face behavioural support for South Asian ST cessation)
3. A combination of Interventions A and B
4. Usual care (VBA).

**Table 1:** Schematic diagram for 2×2 factorial trial of interventions A (NRT) and B (BISCA)

	Intervention A NRT (8 weeks, 4/6 mg)		
**Intervention B -** BISCA		**Negative**	**Positive**
	**Negative**	**No intervention** VBA + self-help material *Trial arm 1*	**Intervention A only** 8-week NRT in addition to standard VBA + self-help material in arm 1 *Trial arm 2*
	**Positive**	**Intervention B only** Behavioural support intervention for ST cessation-BISCA (incorporates VBA and self-help) *Trial arm 3*	**Interventions A and B** *Trial arm 4*

*BISCA* behavioural support intervention for smokeless tobacco cessation in adults, *ST* smokeless tobacco, *VBA* very brief advice, *NRT* nicotine replacement therapy

The factorial design is generally considered to be more efficient than parallel arm trials, as it allows the simultaneous investigation of the effects of two or more treatments, as well as the effects of both in combination [20].

### Study settings

This pilot trial will be conducted in Bangladesh, India and Pakistan which are the three South Asian countries with the highest regional prevalence of ST use. It will be conducted in an urban setting in one administrative site per country. Trial settings have been identified based on the availability of research infrastructure and the local prevalence of ST use. Settings meeting these criteria were identified in Mirpur and Pallabi areas located in Dhaka (Bangladesh), the Essa Nagri Union Council in Karachi (Pakistan), and in Noida, adjacent to Delhi (India). All settings are predominantly low-resource settings with a high population density.

### Study participants and eligibility criteria

The trial aims to recruit adult, exclusive users of ST products who are interested in making a quit attempt. We will screen individuals to identify individuals who (i) use ST products on a daily basis, (ii) are aged 18 years or above, (iii) able to provide informed consent and (iv) motivated and willing to quit ST in the next month. We define daily use as self-reported ST use on at least 25 days in a month over the past 6 months [21].

We will exclude (i) dual tobacco users who either self-report combustible tobacco product use (cigarettes, bidis, hookah) in the past month, or those having a carbon monoxide (CO) level greater than 10ppm on a breath test [22], measured using the Bedfont piCO™ smokerlyzer, (ii) individuals who are currently receiving treatment for tobacco cessation, (iii) individuals whose circumstances might contra-indicate NRT use, such as pregnancy and/or breastfeeding women, and (iv) those reporting unstable episodes of angina pectoris or myocardial infarction or stroke in the past 3 weeks [23].

### Details of interventions

As described above, the trial participants will receive Nicotine replacement therapy (Intervention A) or BISCA (Intervention B), either alone or in combination. The participants not randomised to receive these interventions will receive usual care in the form of VBA [24] offered by a trained cessation advisor. The VBA will last approximately 1–1.5 min. It incorporates the 3As approach (Ask, Advise, Act) previously used for delivering smoking cessation in healthcare settings [24]. During this brief interaction, the advisor will (i) ask about the type of ST product consumed, (ii) identify ST related harms and advise on stopping its use and (iii) act by providing a self-help leaflet to prompt quit planning. Participants receiving VBA will have no further interactions with the cessation advisor. VBA will also be offered to other ST using individuals who are either ineligible for trial participation or those who do not provide informed consent. Details of interventions A and B are provided in further detail below:

#### Intervention A: Nicotine replacement therapy

NRT will be provided to trial participants alongside VBA and the self-help leaflet (described above). Following randomisation, the participants will undertake an 8-week course of NRT chewing gum (Nicorette or its generic equivalent, as per availability in each country); they will receive either 4mg or 6 mg NRT supplies depending on their baseline assessment. A cessation advisor will assess each participant’s tobacco use through two items of the heaviness of Tobacco Use Index [25]: (i) time to first ST use and (ii) average number of daily ST intakes (chews/dips). All participants will receive a standard 4mg dose; however, those who report their first ST intake within 30 min of waking, or consuming ST >10 times/day) will receive a 6-mg dose. The 6-mg dose will be given as a combination of 4-mg and 2-mg nicotine chewing gums taken together.

NRT will be provided by the cessation advisor at the time of recruitment. The participants will be instructed to start using NRT on an hourly basis, up to 15 doses in a day starting from their planned quit date. The first dose will be taken within the hour of waking or as soon as craving develops. They will be instructed to perform the ‘park and chew’ method [26], which involves chewing the gum until a peppery or flavoured taste emerges, ‘parking’ it between cheek and gum to facilitate nicotine absorption through the oral mucosa for approximately 30 min or until taste dissipates. A leaflet providing this information in written form will also be provided.

All participants will be provided with an initial 2-week NRT supply following randomisation; subsequent two weekly supplies of NRT will be provided by a trained research officer to participants who report and demonstrate consumption of NRT on at least 5 days in the previous week. Any adjustments to participants’ NRT dosing, if necessary, will be made in light of participants’ experiences in the first week of its use. Where available, participants will be provided the option to choose alternate NRT flavours in order to maximise their adherence to treatment.

#### Intervention B: BISCA

**Fig. 1:**
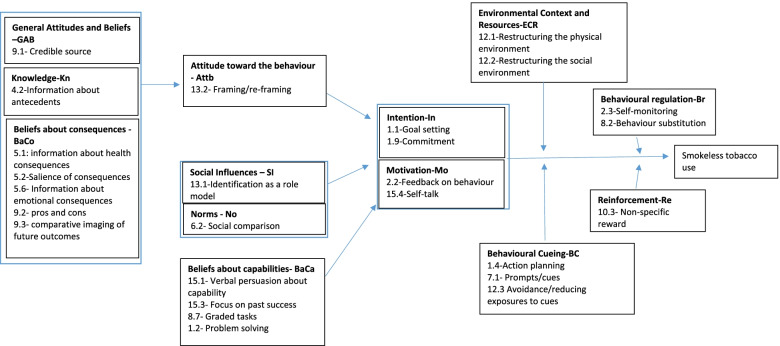
The BISCA logic model identifying potential mechanisms of action (MoAs) and their underlying behaviour change techniques (BCTs)—MoAs are identified in bold text in each box, with related BCTs listed underneath

BISCA is a face-to-face behavioural support intervention that will be provided to trial participants. BISCA is a theory-based, culturally modified intervention that has previously been developed and tested among South Asian ST users in the UK and Pakistan, and adapted for wider testing in Bangladesh, India and Pakistan. BISCA comprises a set of 23 activities that target evidence-based behaviour change techniques (BCTs) that attempt to change ST behaviour through modifying the underlying mechanisms of action (MoA’s) (Fig. 1, BISCA causal model). Intervention resources consist of an advisor flipbook, a client booklet and a self-help calendar for ST users. The delivery of BISCA is structured into pre-quit, quit and post-quit sessions which are delivered by trained cessation advisors. The flipbook resource contains a set of slides presenting a set of scenarios and incorporating photographs for advisors to initiate contact and dialogue with participants. The client pack, a take-home resource given to participants, comprises the client booklet and an 8-week, self-help calendar. The client booklet reinforces the key messages delivered in the face-to-face session while the calendar prompts participants to self-monitor and record their ST use over time.

The participants receiving BISCA will be scheduled for an initial (pre-quit) contact session on the day of randomisation or on another convenient day in the same week. Activities delivered in this session focus on building knowledge around the ST ingredients and harms related to ST use, building self-efficacy and preparing participants for making a quit attempt. The participants will receive one pre-quit session prior to the quit date; however, those who are unable to proceed with a quit attempt will be eligible to receive an additional pre-quit session.

The quit session will be scheduled in the week of the participants’ quit date (either on the same day or 2–3 days preceding the quit date). Each participant will receive one quit session which will focus on strengthening their ex-user identity, identifying triggers and withdrawal symptoms and discussing strategies to manage these. They will also be provided with a self-help calendar on this day for monitoring progress and setting self-incentives.

After the quit session, the participants will receive six weekly post-quit sessions with their cessation advisor. These sessions will focus on the reinforcement of key messages delivered in pre-quit and quit sessions, as well as support in avoiding relapse and minimising withdrawal effects.

The pre-quit session will be conducted at the recruitment centres, while the quit and post-quit sessions will either be delivered at the same place or in participants’ homes. The sessions will be scheduled in advance, and a reminder will be issued to participants ahead of the visit. Those who are unable to attend or have to cancel their scheduled visit will be rescheduled on a convenient date within the same week. A log of delivered sessions will be maintained by the advisors to record the total number of contacts made with each participant. The participants who report lapses on two consecutive weeks will receive no further post-quit sessions; however, they will be provided VBA to plan and execute their next quit attempt. The participants who are non-responsive to phone calls or home visits on three separate occasions will be considered as being lost to follow-up and continuing their ST use.

### Identification and screening of trial participants

The trial participants will be identified from health facilities located in the respective study locations in each country. These are two NGO-based primary healthcare facilities in Mirpur/Pallabi (Bangladesh) and two primary care clinics based in Essa Nagri (Karachi). The selected facilities were identified from a list of primary care and general practices which, following assessment, were selected by country trial teams for the feasibility of conducting research activities. In Noida (India), eligible participants will be identified through health and well-being clinics that offer general health promotion and screening services. In order to maximise the identification and recruitment of participants through each of these facilities, country teams will undertake community mobilisation using a range of techniques in catchment areas. These techniques include public announcements, distribution of brochures and information leaflets, as well as the promotion of the trial at health facilities ahead of the trial start. Participants seeking primary care or those self-referring themselves to the health facilities following community mobilisation will be screened for eligibility. The screening process will be carried out by a research officer, who will administer a screening checklist to determine eligibility and a CO breath test to rule out concomitant smoking. A daily log of screening activities will be maintained at each health facility which will be used to record the number of individuals assessed and their eligibility for the trial. For non-eligible individuals, the reasons for ineligibility will be recorded.

### Recruitment of trial participants

Eligible individuals will be provided with an information sheet detailing (a) the purpose of the trial and the study procedures; (b) potential benefits and risks of participation; (c) data handling procedures for privacy and confidentiality; (d) participants rights, including the right to discontinue participation at any time in the trial; and (e) relevant contact details for those who wish to seek more information or clarity on the study. They will be provided sufficient time and opportunity to clarify their queries with the research officer and to include their peers or family members in consideration of trial participation.

An informed consent process will be followed during recruitment. Eligible individuals will read the information sheet or have it read out to them if they are unable to read. They will then be asked to sign the consent sheet or alternatively provide a thumb impression to indicate their consent in the presence of an impartial witness. A copy of the signed consent form will be provided to all trial participants for their records.

### Randomisation and treatment allocation

A centrally prepared computer-generated randomisation sequence will be used to randomly allocate eligible and consenting trial participants to interventions. The sequence will be created using software R v3.4 [27] and enable country-stratified, permuted block randomisation with varying block sizes to be carried out. Eligible participants will be assigned on an equal allocation basis to one of the three interventions or usual care. We will additionally stratify recruitment by health facilities in Pakistan and Bangladesh. The intervention assignment codes will be shared directly with the respective country trial coordinators.

The treatment allocation will be concealed in sealed envelopes. For the recruitment of an eligible participant, the field-based research officer will place a phone call to the country coordinating centre, where the country trial coordinator will generate a trial ID by running the pre-specified code in an R file. Both the trial coordinator and the research officer will be unaware of the treatment associated with each trial ID. Once the trial ID has been provided, a concealed envelope with the corresponding trial ID will be opened to reveal the participant’s allocation. As this is a pilot trial, the objective is not to conceal to either participants or researchers an allocation to a particular intervention. The above process will ensure, however, allocation concealment up until the point of delivery.

### Data collection and follow-up procedure

After recruitment, enrolled trial participants will complete an interviewer-administered Case Report Form (CRF) at baseline. Collected data will include participants’ socio-demographic and household information, current tobacco use, mood and physical symptoms, nicotine dependence, contact with health providers, attempts to quit ST, motivation to quit ST and mediators of ST cessation. These assessments will also be repeated at 6, 12 and 26 weeks following their quit date (Table 2).

**Table 2:** Schedule of research activities

Activity/assessment	***Stage of study***								
	Study member	Recruitment		Intervention			Follow-up		
		Pre-enrolment	Post-enrolment	Pre-quit	Quit	Post-quit	6 weeks	12 weeks	26 weeks
		Screening & consent	Baseline /randomisation	Pre-quit session(s) (BISCA)	Quit session (BISCA)	6 Weekly BISCA sessions	Follow-up 1	Follow-up 2	Follow-up 3
Screening log	Research staff	X							
Eligibility form	Research staff	X							
Informed consent	Research staff	X							
Random allocation	Research staff		X						
Baseline CRF	Research staff		X						
Health resource use Questionnaire	Research staff		X						X
Quality of life assessment (EQ-5D-5L)	Research staff		X						X
VBA for ST cessation	Advisor		X						
BISCA sessions	Advisor			X	X	X			
Adverse/serious adverse events checklist	Research staff/advisor				X	X	X	X	
Follow up CRF	Research staff						X	X	X
Salivary cotinine (biochemical verification)	Research staff		X					X	X
CO assessment	Research staff	X	X					X	X
Qualitative interviews for process evaluation	Research staff						X		

*BISCA* behavioural support intervention for smokeless tobacco cessation in adults, *CRF* case-report forms, *CO* carbon monoxide, *ST* smokeless tobacco, *VBA* very brief advice

Health resource utilization and quality of life (using the EQ-5D-3L instrument [28]) will be measured at baseline and 26-week follow-up as part of a preliminary economic assessment. Taking healthcare provider and patient perspectives, health resource use will include the costs of delivering the specified intervention in each arm (including staff time, materials and logistics), any health service use (including visits to a healthcare provider, primary or tertiary care facility) and any out-of-pocket costs (including travel costs, fees paid to healthcare providers and costs of medicines including any smoking cessation pharmacotherapy bought over-the-counter).

Saliva samples for biochemical verification of tobacco abstinence will be collected from all participants at baseline and those self-reporting abstinence to all forms of tobacco at 12 and 26-week follow-ups. Self-reported continued NRT use at these follow-ups will delay the collection of a saliva sample until participants report its use as discontinued. A CO breath test will also be administered at these time points to these participants to rule out any smoked tobacco use. The saliva samples from Bangladesh and Pakistan will be transferred to a specialist laboratory (ABS laboratories, York, UK) for biochemical analysis for salivary cotinine while those collected in India will be analysed locally through an accredited laboratory.

### Outcomes

The following will data will be analysed to address the primary (feasibility) objectives of the trial:

#### Recruitment, randomisation and retention

- Number of participants screened, eligible and successfully recruited into the trial, along with characteristics of non-consenting and ineligible participants.
- Total number of participants enrolled in the allocated recruitment period and time to complete recruitment in each country.
- Retention of trial participants in their original trial arms following randomisation and the proportion of participants attending data collection follow-ups at 6, 12 and 26 weeks along with number and reasons for dropping out.

#### Intervention delivery

- The proportion of participants attending the pre-quit and post-quit sessions, the number of quit and post-quit sessions conducted per participant.
- The proportion of trial participants receiving low (4mg) vs high (6mg) dose NRT, the proportion of participants retaining their medication charts and demonstrating their adherence to NRT at follow-up visits.

#### Data collection methods

- Proportion of completed baseline and follow-up assessments in each arm
- Total number of participants eligible to provide saliva samples at 12 and 26 weeks and number of trial participants successfully providing a saliva sample.

The following data will be collected and analysed to address part of the secondary objectives, i.e. the identification of cessation outcomes for the main trial:

- A 7-day point prevalence of tobacco use (smoked and smokeless) at 6 weeks, 12 weeks and 26 weeks of follow-ups. At 26 weeks, the point prevalence will be verified by CO breath test (<10ppm) and salivary cotinine assessment (<15ng/ml).
- Continuous abstinence to all forms of tobacco at 26 weeks post-quit date. This will be self-reported only, as we did not attempt to validate abstinence every week.

Other aspects of the secondary objectives such as process evaluation and health economic procedures are described in subsequent sections.

### Sample size

We calculated the sample size in line with recommendations by Viechtbauer et al. [29], which state that the sample size should be sufficiently large to capture at least one incidence of a range of anticipated and unanticipated outcome events. Assuming the probability of these outcome events to lie between 5 and 10%, we estimated at least 60 participants in each country, using a confidence level of 95%. We increased this number to 80 participants per country in order to have at least 20 participants per trial arm and further inflated this figure by 10% to account for potential loss to follow-up. In total, we thus require 88 participants per country (22/trial arm) with a combined total of 264 participants in Bangladesh, India and Pakistan.

### Statistical analysis

Descriptive data analyses will be performed to describe participant characteristics, as well as to address the feasibility objectives identified above. Reporting will be done for the overall sample and where relevant, stratified for each country. Measures will include summary statistics such as mean and standard deviation for continuous variables and frequencies (absolute and relative) for categorical variables. We will estimate risk ratios and 95% confidence intervals for the potential cessation-related outcomes as a preliminary assessment of effect. No inferences however will be made based on these results. All statistical analysis will be performed on STATA version 16 [30].

### Process evaluation

The process evaluation will be informed by the Medical Research Council guidance [31]. This identifies three components: implementation, mechanisms of impact and context.

#### Implementation and context

One to 2 cessation advisors will be interviewed in each country to explore their experiences of delivering the BISCA and NRT interventions. The impact of factors such as the community environment and other social, economic, cultural, environmental and political factors will be assessed. Fidelity to delivering the BISCA/NRT interventions will be assessed using a fidelity index adapted from the original BISCA study [18]. This will be applied to audio-recordings of nine randomly selected participants per country (3 per intervention arm, excluding those allocated to receive VBA) who have completed their allocated intervention.

#### Mechanisms of impact

All participants will complete a short questionnaire at 6-week follow-up which will have some acceptability questions on trial processes. The questionnaire will also explore the engagement with BISCA and NRT intervention components, acceptability of the sessions and perceived benefits/dis-benefits. A purposive sample of 24 participants in each country (8 from each intervention trial arm), a mix of men and women who have/have not quit ST will be interviewed to explore key issues that emerged in the questionnaire. Data on potential mediators (see Fig. 1) will be collected at all time points.

Interviews will be conducted using a topic guide and digitally audio-recorded. The quantitative data from the fidelity index and questionnaire will be analysed using descriptive statistics. The qualitative data from the questionnaire will be analysed using content analysis [32], while transcribed and translated interviews will be analysed using the Framework Approach [33]. Micorosoft Excel will aid data handling. The integration of interview findings with respective questionnaire data will be done using a ‘triangulation protocol’ [34].

### Health economics

The analysis of economic data will include a preliminary assessment of the costs associated with implementing BISCA (alone or in combination with NRT) and NRT alone as well as a change in EQ-5D-3L scores between the baseline and 26 weeks post-baseline. The overall assessment will include a commentary on the suitability/feasibility of an economic evaluation alongside a future definitive trial.

### Data management

Each country site will hold data in accordance with both the UK (General Data Protection Regulation [35], Data Protection Act 2018 [36] and any additional country-specific requirements. Collected data will be identified by a unique identification number (i.e. the Trial ID number) only. Study files will be stored in accordance with UK Good Clinical Practice (GCP) guidelines [37]. The collected data will be stored separately from the consent sheets to maintain the anonymity of data. Research staff at the University of York will develop and maintain an electronic database that will be used to enter data by research staff in each participant country. The database will be uploaded to a secure server provided by York University IT services and accessible only to researchers directly involved in the study or by representatives of the Trial Data Management and Ethics Committee in the event of a study audit. Similarly, all audio files and anonymised transcripts will be uploaded and kept secure throughout the duration of the study. Paper versions of study documents at the participating sites, the country coordinating centers and the University of York will be retained in a secure (locked when not in use) location, during and after the trial has finished. All essential documents, including source documents, will be retained for a minimum period of 5 years after study completion.

### Trial monitoring procedures

Mechanisms for trial monitoring include collection and reporting of adverse events, regular trial audits and safety monitoring by an independent Data Management and Ethics Committee (DMEC).

#### Adverse events

A standardised approach for adverse events will be followed for their identification, recording and reporting. Checklists will be completed each week, either face-to-face or via a phone call, to assess the frequency and severity of Adverse (AEs) and Severe Adverse Events (SAEs). All participants will also have access to a central number to report any sudden onset AEs. Reported AEs will be assessed by the country trial coordinator for seriousness, severity and relatedness to the intervention. AEs related to the intervention will also be assessed for their expectedness. Those with the potential to become SAEs will be monitored till resolution. SAEs which are related to the intervention will be reported to the DMEC and Trial Steering committee within 3 days and the local Ethics Committees within 15 days. The participants who experience serious unexpected AEs related to the intervention will be withdrawn from treatment but will continue to be followed up in their respective trial arm.

#### Trial audit

Trial coordinators in each country will carry out regular monthly audits, using monitoring checklists, to ensure all staff are complying with standard operating protocols (SOPs) for implementation of all trial procedures and that appropriate documentation is held at each trial site. In addition, a lead investigator will make at least one planned visit to each country’s coordinating centre during recruitment and follow-up. Additional unplanned visits by the trial coordinators may also be triggered if specific concerns are raised by the trial management team in order to address any potential issues around data queries, compliance with the trial procedures or any other logistic issues at trial sites.

#### Data monitoring and ethics

An independent DMEC, consisting of an independent trial statistician and two subject experts, will oversee progress in recruitment, safeguard the interests of trial participants, assess the safety and futility of the interventions during the trial and monitor the overall conduct of the trial. The DMEC will convene around recruitment milestones and will provide recommendations and advice to the Trial Steering Committee and Trial Sponsor on the basis of submitted reports. Possible recommendations include the following:

1. No action is needed, the trial continues as planned.
2. Early stopping due, for example, to clear harm of treatment, futility or external evidence.
3. Extending recruitment activity to additional sites in countries with low recruitment.
4. Stopping or suggesting a modification to any arm of the trial.
5. Sanctioning and/or proposing protocol changes in line with patient safety.

### Dissemination of results

The trial results will be relevant to a wide range of academic audiences interested in tobacco cessation in low- and middle-income countries. These include (i) academics, (ii) clinicians, (iii) graduate and post-graduate students, (iv) health economists and (v) researchers working in behavioural sciences, tobacco control and global public health. The primary means of dissemination to these audiences will be the publication of trial findings in open-access scientific journals. Each manuscript will be developed in line with ASTRA’s publication policy, which also defines authorship criteria based on the ICMJE guidelines [38] and entered into ASTRA’s publication log maintained at the University of York. In addition, the results will be disseminated at international conferences and seminars on tobacco, oral health, cancer and non-communicable diseases.

Non-academic audiences include two groups. The first group includes people directly involved in research, i.e. research participants, their families and community stakeholders (residents, local administration, health workers working in the respective communities). A dissemination workshop will be organised for these audiences at the end of the trial highlighting key findings and results. The second group includes a wider group of stakeholders—the general public, physicians, policymakers, tobacco control advocates and non-governmental organisations. In each country, stakeholder forums including these groups have been established as part of ASTRA’s objectives. We will communicate trial findings to these stakeholders in planned annual meetings. Besides these avenues, dissemination will also be carried out through electronic, print and/or social media on events such as World No Tobacco Day and World Cancer Day. Findings will also be shared through social media platforms (ASTRA Twitter account @ASTRA_NIHR), the University of York ASTRA webpage (https://www.york.ac.uk/healthsciences/research/public-health/projects/astra/), as well as through webpages of ASTRA’s partner institutions and Global health groups such as RESPIRE [39, 40].

## Discussion

The ASTRA feasibility study responds to the urgent need to identify culturally suitable, evidence-based interventions for supporting successful quit attempts among ST users in the South Asian region. Ongoing evaluation and broader future implementation of such interventions is highly likely to yield health-related and economic benefits to both ST users and the wider society.

Our approach proposes a combination of two evidence-based interventions, implemented using a robust study design and validated outcome measures. The primary focus of this present study on feasibility will identify implementation-related issues of relevance to developing the main trial. These may include a general reluctance towards participation, collection of saliva samples and potential for loss to follow-up due to participant migration/dispersion. The secondary focus on effectiveness will make a contribution to the developing evidence base on ST cessation, particularly in the South Asian region. The small sample size compromises the assessment of effectiveness in this study, which is a natural limitation of feasibility trials. Another limitation may be the long-term availability and provision of pharmacotherapy which is essentially an ‘out-of-pocket’ expense in the South Asian region. This financial barrier has to be addressed through ASTRA policy-oriented initiatives.

### Protocol amendments

The current version of the trial protocol (version 1.2.1 dated on 21 February 2020) incorporates changes recommended by the trial sponsor and the Trial Management Team to date (supplementary file 1—protocol amendments). All amendments to the protocol are first discussed with the Lead Investigator and then submitted to the Research Governance Committee for formal approval. A judgement is made on the nature of the amendment, i.e. major or minor, applying guidance from the University of York Research Governance Committee. All minor amendments are implemented once notified, while all major amendments are implemented once approved by the respective national ethics committees in each country.

## Supplementary Information

**Additional file 1.** SPIRIT 2013 Checklist: Recommended items to address in a clinical trial protocol and related documents.

## Data Availability

Not applicable.
